# COVID-19 Impacts Across Multiple Life Domains of Vulnerable Socio-Demographic Groups Including Migrants: A Descriptive Cross-Sectional Study

**DOI:** 10.3389/ijph.2022.1604665

**Published:** 2022-05-11

**Authors:** Felix P. Chilunga, Liza Coyer, Didier Collard, Tjalling Leenstra, Henrike Galenkamp, Charles Agyemang, Maria Prins, Karien Stronks

**Affiliations:** ^1^ Department of Public and Occupational Health, Amsterdam UMC location University of Amsterdam, Amsterdam, Netherlands; ^2^ Public Health Department, Amsterdam, Netherlands; ^3^ Centre for Urban Mental Health, University of Amsterdam, Amsterdam, Netherlands

**Keywords:** social determinants of health, COVID-19, impact, long-term conditions, vulnerable populations, migration, ethnic minority

## Abstract

**Objectives:** We assessed the impacts of COVID-19 on multiple life domains across socio-demographic groups in Netherlands.

**Methods:** After the first COVID-19 wave, we distributed online questionnaires among 13,031 participants of the multi-ethnic HELIUS cohort. Questionnaires contained questions on changes in income status, healthy behaviors, mental health, and access to non-COVID-19 health care. We then calculated differences in adjusted proportions of participants that reported negative changes across multiple life domains by migration background, age, sex, education, and occupation.

**Results:** 4,450 individuals (35%) responded, of which 4,294 were included. Older populations and men seemed to be less vulnerable to negative changes in multiple life domains during the COVID-19 pandemic as compared to the pre-pandemic period, while populations with a migration background and lower education/occupation groups seemed to be more vulnerable to negative changes.

**Conclusion:** Not all populations vulnerable to SARS-CoV-2 infection and mortality are also more vulnerable to COVID-19 impacts across multiple other life domains. Targeted interventions are needed in socio-demographic groups that are most impacted by COVID-19 in various life domains to prevent a further increase of their already increased risk of chronic diseases after the pandemic.

## Introduction

The acute and direct impacts of COVID-19 on an individual’s health are well known in terms of morbidity and mortality [1], as well as by the vulnerable socio-demographic groups that are likely to suffer these consequences [2–6]. For instance, ethnic minority groups residing in high income countries, lower socio-economic status groups and men are at greater risk of SARS-CoV-2 infection and subsequent COVID-19 related morbidity and mortality [2–5]. Additionally, young people are more likely to get infected, while older persons have the highest morbidity and mortality from the disease [6].

Beyond these direct health impacts on an individual, COVID-19 and its prevention measures (e.g., lockdowns) also affect multiple other domains of life including family and social life, social relationships, health behaviors, mental health, employment, health services and social care use, quality of care received [7–10]. As a matter of fact, issues in these life domains are also determinants of negative health consequences (i.e. long term drivers of health) [11]. For example, unhealthy behaviors such as poor diet, tobacco smoking and physical inactivity, as well as psychosocial stress, are risk factors for non-communicable diseases [10, 12]. Changes in multiple life domains due to COVID-19 therefore has bearing on future health consequences in an individual and the society at large. This will need urgent addressing to promote a healthy population post-COVID-19 pandemic.

Although a previous study in the UK recently showed that ethnic minority groups, groups with low education and women reported most changes in sleep, exercise and diet, the study was only limited to these behavioral factors and changes in many other life domains across socio-demographic groups are still unknown [13]. Identification of population groups that are most vulnerable to the impacts of COVID-19 across multiple life domains will therefore help in development of strategies that curb negative future health consequences well before they become apparent.

Based on observations that the risk of SARS-CoV-2 infection across socio-demographic groups results from clustering of various factors (e.g., working in the front line, household size, etc.) [14], we hypothesize that the impacts of COVID-19 on multiple life domains will also be more pronounced in socio-demographic groups that are most vulnerable to infection. This means that these vulnerable socio-demographic groups will not only suffer from acute impacts of COVID-19 on health, but also the negative health consequences well after the pandemic. We, therefore, assessed the impacts of COVID-19 on multiple life domains (i.e., income status, healthy behaviors, mental health, and use of non-COVID-19 health care) across socio-demographic groups (i.e., migration background, age, sex, education, and occupation) in Netherlands.

## Methods

### Study Design and Population

The current study was conducted as part of the Healthy Life in an Urban Setting (HELIUS) study [15, 16]. The HELIUS study is a multiethnic cohort study initiated in 2011 in Amsterdam (Netherlands) focusing on cardiovascular diseases, mental health, and infectious diseases. A full description of the cohort is provided elsewhere [15, 16]. In brief, HELIUS included a total of 24,789 persons of the Dutch, South-Asian Surinamese, African Surinamese, Ghanaian, Moroccan, and Turkish origins, aged between 18 and 70 years at inclusion. Participants were randomly sampled from the municipality register of Amsterdam by migration background (immigrants and their descendants). All participants completed a self-administered questionnaire and underwent a physical examination during which biological samples were obtained.

Migration background was based on the standard classification of Statistics Netherlands [17]. This standardized classification considers the country of birth of residents and their parents, thus includes immigrants’ descendants [17]. Participants are considered of Dutch origin if; 1) they were born in Netherlands, and at least one parent born was also born in Netherlands or 2) they were born abroad but both their parents were born in Netherlands. On the other hand, participants were considered as immigrants and their descendants if; 1) they were born abroad and had at least one parent born abroad (immigrants) or 2) they were born in Netherlands, but both their parents were born abroad (immigrants’ descendants). Participants of Surinamese origin were further classified as African Surinamese origin, South-Asian Surinamese origin, and Javanese/other/unknown Surinamese origin, based on self-report.

### Ethical Approval and Informed Consent

Ethical approval for the HELIUS study was obtained from the Academic Medical Center Ethical Review Board. All participants provided written informed consent.

### COVID-19 Sub-Study

Between 27 August 2020 and 29 September 2020 (after the first COVID-19 wave in Netherlands), 13,031 HELIUS participants with email addresses were invited to participate in an online COVID-19 sub-study. One of the goals of the sub-study was to understand the impact of COVID-19 on wellbeing and use of non-COVID-19 health care. Participants were invited to complete an online questionnaire which was adapted from Netherlands Institute for Public Health and the Environment (Supplementary Appendix S1) [18]. The questionnaire was available in Dutch, English and Turkish. Among the questions were those on the changes in finances, health behavior, mental health factors, and use of non-COVID-19 health care due to the COVID-19 pandemic (Supplementary Appendix S1). Participants could in turn provide a response from a set of options based on the Likert scale (Supplementary Appendix S1). To mitigate non-response that arises from completing very long questionnaires [19], our questionnaire was split into four sections, whereby each participant responded to only one section of the questionnaire.

### Other Measurements

Information on socio-demographics was obtained from the main HELIUS study database at baseline. They were categories as follows; age into <40 years old (younger age), 40–65 years old (middle age), >65 years old (older age); sex into male and female; migration background into Dutch origin, African Surinamese origin, Ghanaian origin, Moroccan origin, South-Asian Surinamese origin and Turkish origin; migration generation into immigrants, and their offspring; education into never been to school or elementary school, lower vocational or secondary school, intermediate vocational or secondary school, and higher vocation school or university; occupational status into elementary, lower, intermediary, higher occupations and scientific occupations. Health literacy was measured using the validated set of brief screening questions (SBSQ) questionnaire [20], and categorized into adequate or inadequate using cut-offs proposed by Chew et al. [21].

### Statistical Analysis

Statistical analyses were performed in R (Version 4.0.2). Summary statistics were presented as proportions. Differences in baseline characteristics were tested with χ^2^ tests. The Adj.prop package was used to calculate the proportion of participants with an outcome of interest adjusted for age and sex. Our outcomes of interest were proportion of participants that responded with the following changes due to the COVID-19 pandemic; 1) “yes” to job loss, 2) “yes” to having trouble with family income, 3) “less” and “much less exercise,” 4) “less” and “much less healthy diet,” 5) “more” and “much more alcohol consumption,” 6) “more” and “much more smoking,” 7) “more” and “much more stress,” 8) “more” and “much more trouble sleeping,” 9) “more and much more lonely,” 10) “agree and agree completely” to reluctance to go to the doctor, to reluctance in allowing care givers into their home, to not receiving professional care, and to being denied care altogether. All analyses were stratified by five socio-demographic factors that have been shown to be increase vulnerability for SARS-CoV-2 infection (i.e., migration background, age, sex, educational level, and occupation status). Adjusted proportions were reported together with their 95% confidence intervals. Due to the descriptive nature of the study and to the smaller sample sizes per group after stratification (low study power), logistic regression analyses were not performed. For instance, an average of 28 Ghanaians responded to each of the four questionnaire parts, which is well below the threshold of 100 participants required to perform subgroup multivariate analyses in observational studies [22]. Moreover, combining the populations with a migration background would not reflect the large socio-cultural differences between these groups. All analyses were two tailed at an alpha of 0.05. Interpretation of results was based on general patterns observed within a socio-demographic category (e.g., general pattern of results in young populations vs. the general pattern of results in older populations) instead of evaluating each outcome individually via confidence intervals.

## Results

**FIGURE 1:**
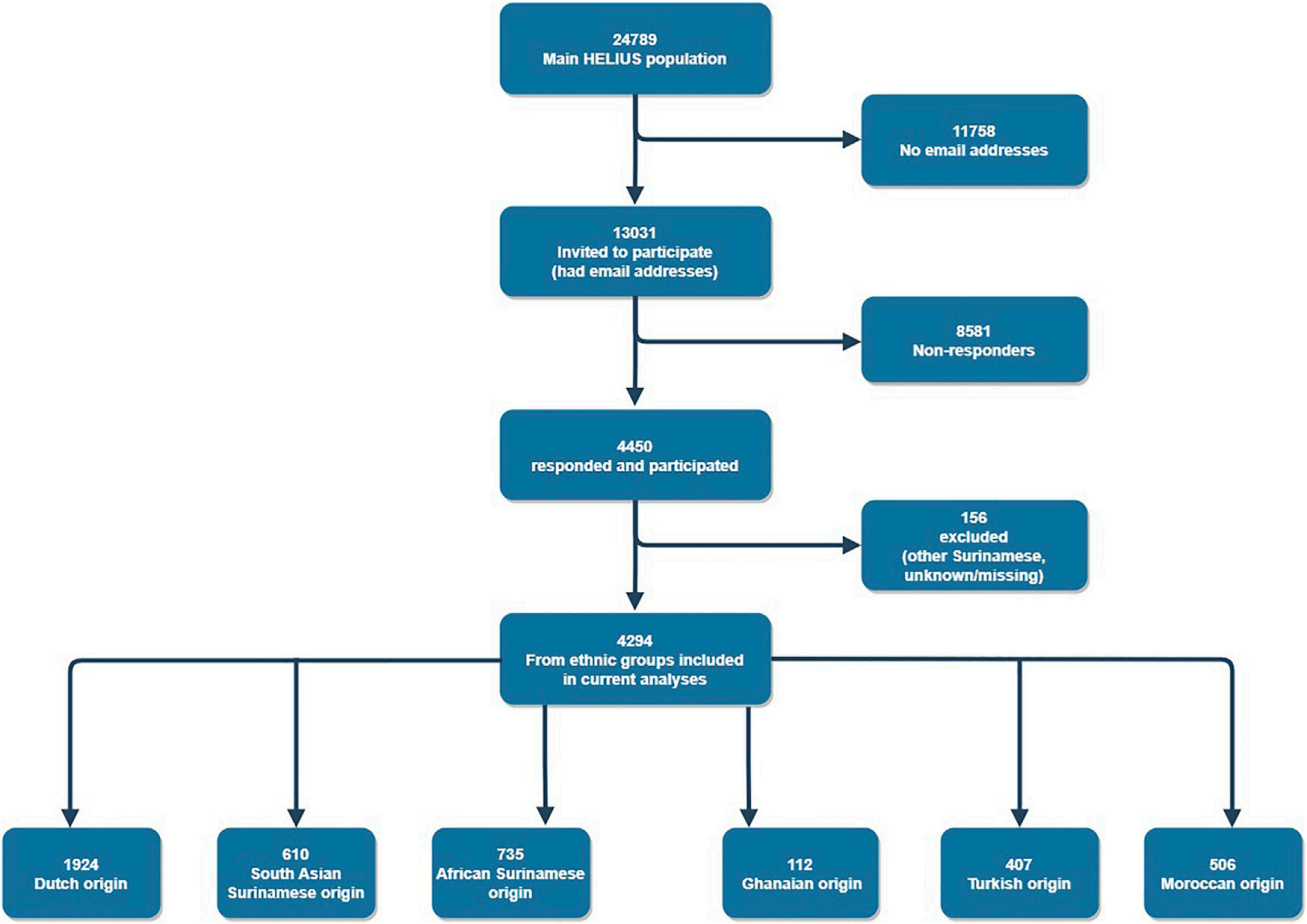
Flow chart of participation in the study (HELIUS study, Netherlands, 2022).

A total of 4,450 individuals responded to the online questionnaire representing a 35% response rate (Figure 1). Our sub-sample was representative of the general HELIUS population except for education and occupation (Supplementary Appendix S2). Specifically, our sample comprised of a higher proportion of participants with higher education and occupations than the general HELIUS population. Section one of the questionnaire was responded to by 1084 (24.3%) participants, section two by 1123 (25.3%) participants, section three by 1138 (25.6%) participants and section four by 1105 (24.8%) participants. The profile of participants was similar across all four sections of the questionnaire ruling out inter-section response biases (Supplementary Appendix S3).

A total of 4,294 participants were included in the final analyses (Table 1). Majority were of Dutch origin (43.2%), while Ghanaian origin populations were least represented (2.5%). Majority of participants were also female (56.5%), middle aged (61.8%) and had adequate healthy literacy (95.7%). Additionally, most participants were educated to university level (45.4%) and had higher occupations (41.9%). Populations with a migration background had mostly migrated themselves (77.4%).

**TABLE 1:** Baseline characteristics by migration background (HELIUS study, Netherlands, 2022).

Characteristic	Total (N = 4294)	Dutch origin (N = 1924)	South-Asian Surinamese origin (n = 610)	African Surinamese origin (*n* = 735)	Ghanaian origin (*n* = 112)	Turkish origin (*n* = 407)	Moroccan origin (*n* = 506)	*p*-value^a^
	n (%)	n (%)	n (%)	n (%)	n (%)	n (%)	n (%)	
Gender								<0.001
Male	1871 (43.6)	856 (44.5%)	262 (43.0%)	253 (34.4%)	60 (53.6%)	205 (50.4%)	235 (46.4%)	
Female	2423 (56.4)	1068 (55.5%)	348 (57.0%)	482 (65.6%)	52 (46.4%)	202 (49.6%)	271 (53.6%)	
Age in years on 1 January 2020								
Median [IQR]	52 [43–60]	58 [47–66]	54 [45–61]	58 [49–65]	53 [41–60]	46 [38–54]	45 [36–54]	<0.001
Age categories (years)								<0.001
<40	809 (18.8)	300 (15.6%)	106 (17.3%)	73 (9.9%)	25 (21.9%)	126 (31.2%)	179 (35.4%)	
40–65	2645 (61.6)	1067 (55.5%)	430 (70.5%)	499 (67.9%)	76 (68.6%)	269 (66.2%)	304 (60.1%)	
>65	840 (19.6)	557 (28.9%)	74 (12.2%)	163 (22.3%)	11 (9.5%)	12 (2.5%)	23 (4.5%)	
Migration generation								<0.001
1st	1805 (43.6)	—	482 (79.0%)	619 (84.2%)	103 (92.0%)	269 (66.1%)	332 (65.6)	
2nd	565 (13.2)	—	128 (21.0%)	116 (15.8%)	9 (8.0%)	138 (33.9%)	174 (34.4)	
Educational level								<0.001
No School/Elementary School	197 (4.6)	43 (2.2%)	28 (4.6%)	11 (1.5%)	12 (10.7%)	44 (10.8%)	59 (11.7%)	
Lower Secondary School	833 (19.4)	212 (11.0%)	180 (29.5%)	198 (26.9%)	48 (42.9%)	98 (24.1%)	97 (19.2%)	
Intermediary Secondary School	1248 (29.1)	389 (20.2%)	218 (35.7%)	268 (36.5%)	32 (28.6%)	142 (34.9%)	199 (39.3%)	
Higher Vocational/University	1984 (46.2)	1272 (66.1%)	184 (30.2%)	255 (34.7%)	18 (16.1%)	115 (28.3%)	140 (27.7%)	
Missing	32 (0.7)	8 (0.4%)	0 (0.0%)	3 (0.4%)	2 (1.8%)	8 (2.0%)	11 (2.2%)	
Professional level								<0.001
Elementary occupations	181 (4.2)	23 (1.2%)	22 (3.6%)	22 (3.0%)	43 (38.4%)	35 (8.6%)	36 (7.1%)	
Lower occupations	788 (18.4)	206 (10.7%)	149 (24.4%)	181 (24.6%)	27 (24.1%)	101 (24.8%)	124 (24.5%)	
Intermediary occupations	1163 (27.1)	424 (22.0%)	218 (35.7%)	257 (35.0%)	12 (10.7%)	107 (26.3%)	145 (28.7%)	
Higher occupations	1250 (29.1)	756 (39.3%)	130 (21.3%)	193 (26.3%)	9 (8.0%)	68 (16.7%)	94 (18.6%)	
Scientific occupations	572 (13.3)	431 (22.4%)	43 (7.0%)	38 (5.2%)	4 (3.6%)	39 (9.6%)	17 (3.4%)	
Missing	340 (7.9)	84 (4.4%)	48 (7.9%)	44 (6.0%)	17 (15.2%)	57 (14.0%)	90 (17.8%)	
Difficulty with Dutch language								<0.001
No	1799 (41.9)	—	511 (83.8%)	681 (92.7%)	34 (30.4%)	231 (56.8%)	342 (67.6%)	
Yes	549 (12.8)	—	99 (16.2%)	53 (7.2%)	77 (68.8%)	166 (40.8%)	154 (30.4%)	
Missing	22 (0.5)	—	0 (0.0%)	1 (0.1%)	1 (0.9%)	10 (2.5%)	10 (2.0%)	
Health literacy (SBSQ)								<0.001
Adequate	4140 (96.4)	1913 (99.4%)	600 (98.4%)	724 (98.5%)	91 (81.2%)	352 (86.5%)	460 (90.9%)	
Low	130 (3.0)	6 (0.3%)	10 (1.6%)	10 (1.4%)	20 (17.9%)	48 (11.8%)	36 (7.1%)	
Missing	24 (0.5)	5 (0.3%)	0 (0.0%)	1 (0.1%)	1 (0.9%)	7 (1.7%)	10 (2.0)	

a *p*-value for differences in baseline characteristics between the ethnic groups. *p*-value obtained via chi-square test for categorical variables, or Kruskal wallis test for median age.

SBSQ, set of brief screening questions.

### Impact of COVID-19 on Multiple Life Domains by Migration Background

**FIGURE 2:**
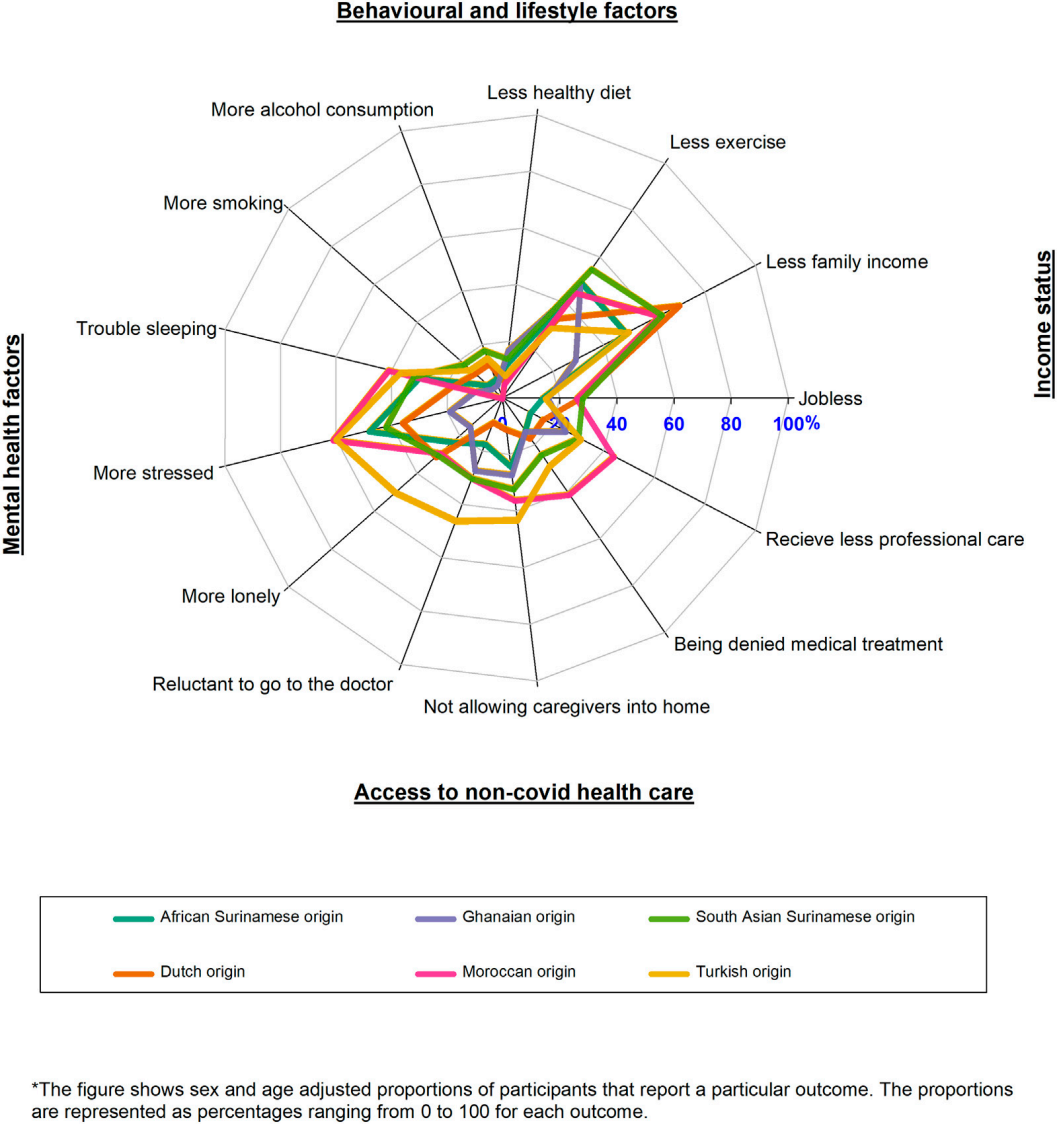
Radar plot of the impacts of Coronavirus disease across multiple life domains by migration background (HELIUS study, Netherlands, 2022).

The Moroccan origin and Turkish origin participants reported more negative mental health factors and more reduction in access to non-COVID-19 health care than the Dutch origin participants during the COVID-19 pandemic as compared to the pre-pandemic period, while the South-Asian Surinamese origin participants reported more unhealthy behaviours than Dutch origin participants during the COVID-19 pandemic as compared to the pre-pandemic period. On the other hand, Dutch origin participants reported more reduction in family income than populations with a migration background during the COVID-19 pandemic when compared to the pre-pandemic period (Figure 2; Supplementary Appendix S4).

### Impact of COVID-19 on Multiple Life Domains Across Age Groups

**FIGURE 3:**
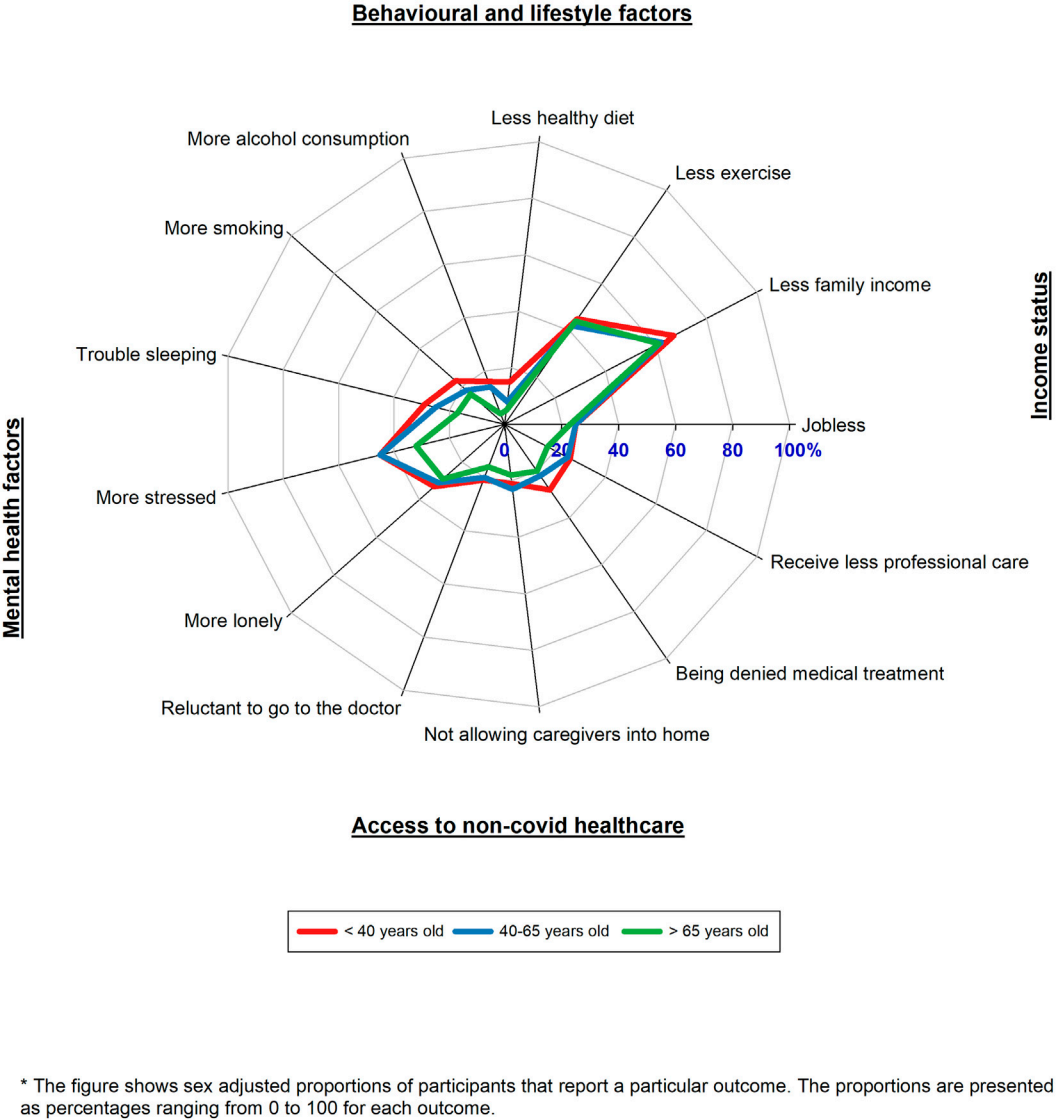
Radar plot of the impacts of Coronavirus disease across multiple life domains by age (HELIUS study, Netherlands, 2022).

Younger participants (<40 years old) reported the largest increase in unhealthy behaviours as well as mental health factors, the most reduction in income, and the most reduction in access to non-COVID-19 health care than older participants (>65 years old) during the COVID-19 pandemic as compared to the pre-pandemic period (Figure 3; Supplementary Appendix S5).

### Impact of COVID-19 on Multiple Life Domains Across Sex Groups

**FIGURE 4:**
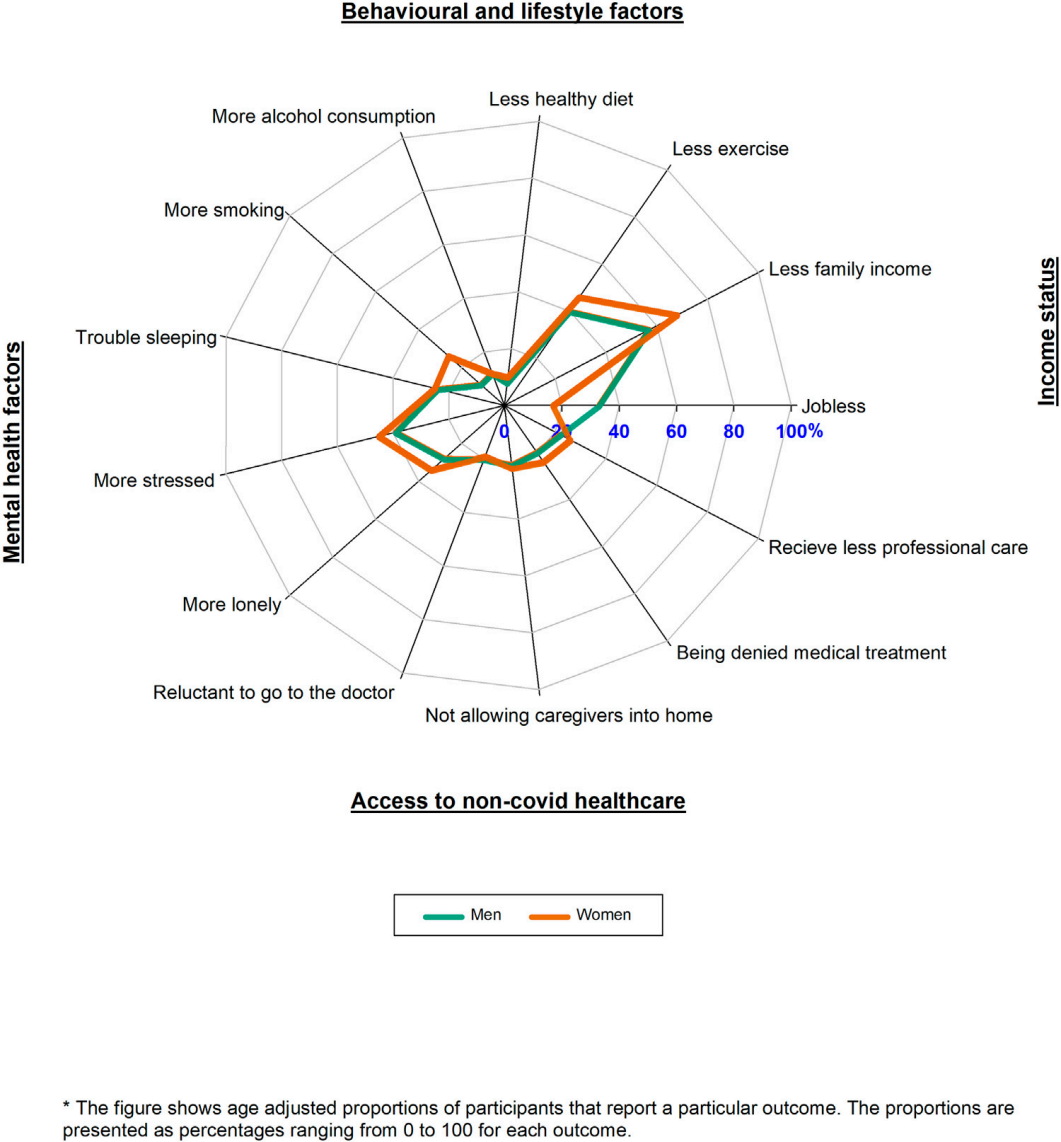
Radar plot of the impacts of Coronavirus disease across multiple life domains by sex (HELIUS study, Netherlands, 2022).

Comparing the COVID-19 pandemic period to the pre-pandemic period, women reported unhealthier behaviours, more negative mental health factors, more reduction in family income, and more reduction in access to non-COVID-19 health care than men. On the other hand, men reported that they were more jobless during the COVID-19 pandemic as compared to the pre-pandemic period (Figure 4; Supplementary Appendix S6).

### Impact of COVID-19 on Multiple Life Domains Across Education and Occupation Levels

**FIGURE 5:**
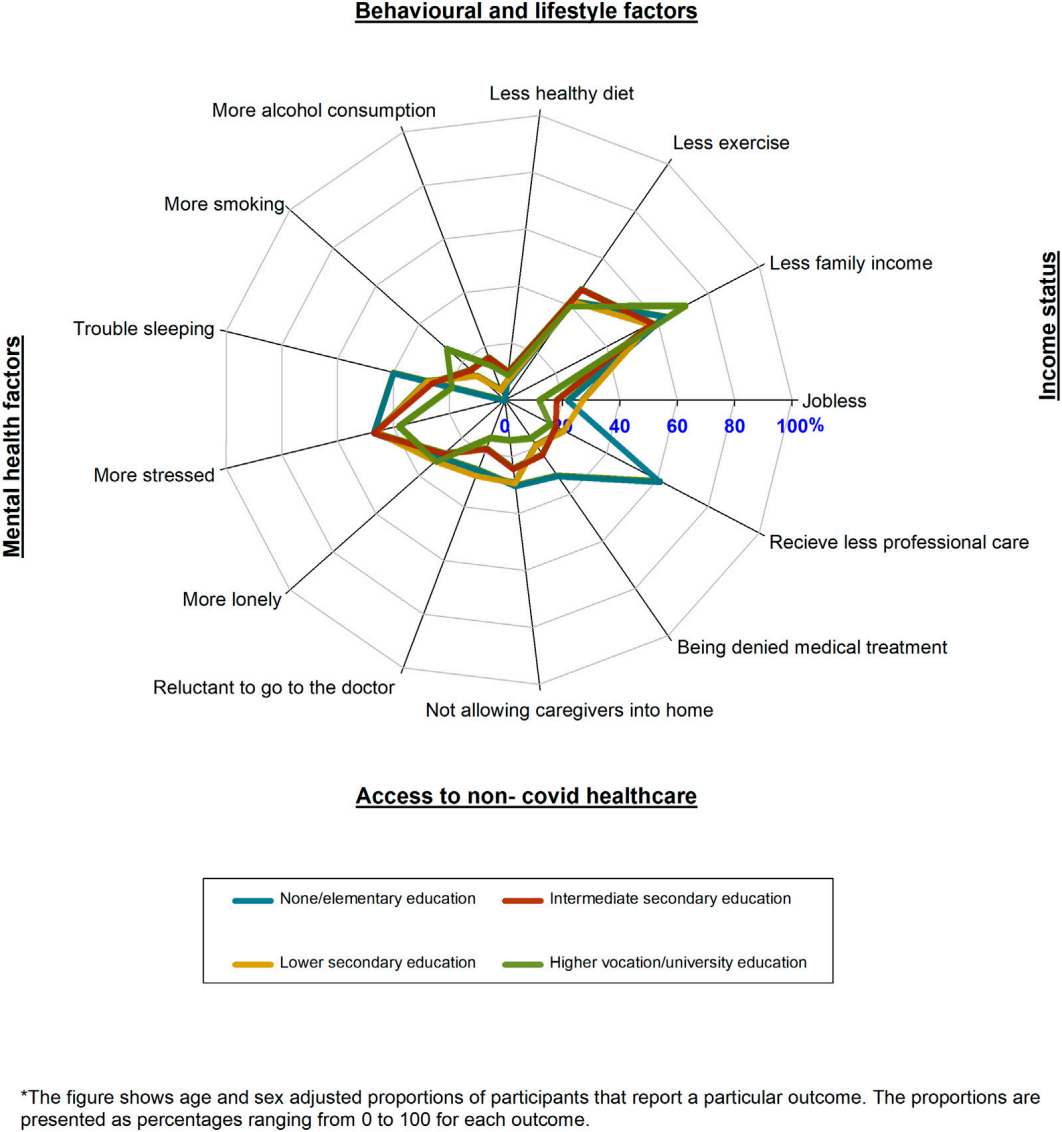
Radar plot of the impacts of Coronavirus disease across multiple life domains by education (HELIUS study, Netherlands, 2022).

Participants with lower education/occupation levels reported more negative mental health factors, more joblessness, and more reduction in access to non-COVID-19 health care than those with higher education/occupation levels during the COVID-19 pandemic as compared to the pre-pandemic period. On the other hand, participants with higher education/occupation levels, reported more unhealthy behaviours than those with no lower education/occupation levels during the COVID-19 pandemic as compared to the pre-pandemic period (Figure 5; Supplementary Appendices S7–S9).

## Discussion

In our study, which was based on descriptive analyses, we found that some population groups that are more vulnerable to SARS-CoV-2 infection, as well as COVID-19 related morbidity and mortality were less susceptible to negative impacts of COVID-19 across multiple other life domains. In particular, older age groups and men seem to be less vulnerable to negative changes in income status, healthy behaviors, mental wellbeing, and access to non-COVID-19 health care. In contrast, populations with a migration background and groups with lower education/occupation showed further vulnerability to COVID-19 impacts across multiple other life domains.

Our results suggest that vulnerability to SARS-CoV-2 infection, as well as COVID-19 related morbidity and mortality among socio-demographic groups does not always extend to multiple other life domains as we had hypothesized. For populations with a migration background, vulnerability to SARS-CoV-2 infection, as well as COVID-19 related morbidity and mortality extended to other life domains. Before the COVID-19 pandemic, previous studies in Netherlands had shown that populations with a migration background had less income [23], more unhealthy diets, less physical activity, more mental health issues and less access to the health care system than the population of Dutch origin [24]. It was therefore not unexpected that this population group also reported/experienced the most negative changes in these life domains during the COVID-19 pandemic. Our findings on behavioural factors are also in line with a study from the UK that showed that ethnic minority groups had more negative changes in sleep, diet and exercise during the lockdown in that country [13]. The actual factors responsible for these changes are beyond the scope of this study, but we postulate that these extensive negative health changes among populations with a migration background would have possibly come about due to various factors that are known to be prevalent in the groups. For instance, the increase in mental health issues (e.g., psychosocial stress and sleeplessness) would have resulted from worrying about the possibility of getting infected or seriously ill or dying due to the high number of infections reported in the group as compared to the population of Dutch origin) [2]. The high burden of chronic health conditions in the group (as compared to the Dutch origin population) would have led to a great reduction in use of non-COVID-19 health care during lockdowns [25]. The respondents of Dutch origin reported the most negative changes in income due to COVID-19. This result was expected as less deprived populations are likely to be more affected by a loss of income compared to those who are more deprived [26]. Moreover, populations with a migration background worked more in the front-line jobs where they still earned money [27], while populations in COVID-19 affected industries/businesses (likely to be of Dutch origin) would have received grants from the Government which were lower than their pre-COVID-19 income leading to a net loss in income [28].

For lower education/occupation groups, vulnerability for SARS-CoV-2 infection, as well as COVID-19 related morbidity and mortality also extended to other life domains. The findings on more negative changes in sleep, exercise and diet in the group as opposed to those with higher education/occupation are also in line with the previous UK study on changes in behavioural factors [13]. Similar to populations with a migration background, the reported increase in psychosocial stresses in lower education/occupation groups would have also resulted directly from the COVID-19 effects (e.g., worrying on the possibility of getting infected/seriously ill/dying due to the high number of infections reported in the group as opposed to highly educated/higher occupation groups) and indirectly from its control measures (e.g., worried on not being able to work from home during lockdowns) [29]. Additionally, lockdown measures and the high burden of chronic conditions in this group (as compared to the highly educated/higher occupation groups) would have negatively influenced physical activity levels and access to non-COVID-19 health care respectively [30].

Younger adult participants (<40 years old) demonstrated vulnerability to changes in many life domains than older participants. Younger populations were more restricted in their daily lives during lockdowns than older populations (e.g., being restricted from going to restaurants) [31]. In fact, younger populations perform more of these activities than older populations. This would have probably led to more mental health issues [32–35], more negative income changes and more unhealthy behaviours (as a possible response to all these stressful events/restrictions) compared to the older age groups [36]. Additionally, older populations have more chronic diseases than the younger age groups [37]. It is therefore possible that older populations were given greater access to the health system due to their chronic health conditions than younger populations. This would have led to a reduction in access to non-COVID-19 health care in the younger populations as opposed to the older populations.

Previous research has shown that there are sex differences in health, including healthy behaviours, mental health, and access to the health care system [38]. Specifically, women are known to have more mental health issues, and more contact with the health system than men [38]. On the other hand, men are likely to have more unhealthy behaviours such as smoking and alcohol consumption than women [39]. Our finding that women had more negative mental health factors, more reduction in family income, and more reduction in access to non-COVID-19 health care due to COVID-19 is in line with the pre-COVID-19 observations. In this case, the COVID-19 pandemic would have negatively exacerbated their mental health (e.g., through women spending more hours teaching children at home during lockdowns as opposed to men) [40]. Additionally, lockdowns would have reduced the contacts of the women with the health care system (as opposed to men who already have less contact with the health care system). Surprisingly, women also had the more negative changes in healthy behaviours (e.g., smoking) compared to men due to the COVID-19 pandemic. This is in contrast to the UK study on behavioural factors that showed that women had less changes in alcohol consumption, exercise and diet [13]. The cause of this increase in unhealthy behaviours in women during the COVID-19 pandemic in Netherlands is not clear and needs further investigation.

Altogether, our findings shed some light on the future health of populations after the COVID-19 pandemic. Our findings show that populations with a migration background, groups with lower education/occupations, younger populations and women are at an increased risk of ill-health after the COVID-19 pandemic. Since explanations for our findings are mainly theoretical and not-exhaustive, further studies should be undertaken to better understand the underlying mechanisms of our observations.

The main strength of our study is that it incorporates a broad range of outcomes in addition to those reported in a previous UK study. Another strength of our study is that it includes both the Dutch origin population and populations groups originating from multiple other countries. An additional strength of the study is that the questionnaire was adapted from the Public Health Institute of Netherlands which increased the reliability of our findings. In fact, the changes due COVID-19 across life domains reported in the Dutch origin population from our study are comparable to those reported in the RIVM study (95% Dutch participants) [18]. On the other hand, our study is not without limitations. First, the analyses were based on self-reports which can be influenced by responder bias. Second, the questionnaire was translated into three languages (Dutch, English and Turkish) and would have possibly presented response challenges to participants who are not proficient in these languages, also leading to response bias. Third, the current sample was comprised mainly of Dutch origin, highly educated, and higher occupation participants than the general HELIUS population. There is a possibility of selection bias from this sample structure, but this type of bias could have been minimised by analysing the data across strata of socio-demographic groups. Fourth, participants responded to different sections of the questionnaire, which can lead to inter-section response biases. Although the profile of responders to each of the four questionnaire sections was similar based on a set of measured characteristics (i.e., migration background, sex, age, education, occupation, Dutch proficiency, health literacy), it is also possible that other unmeasured characteristics (e.g., underlying health conditions) can still introduce selection bias within the groups. Fifth, due to the low power of the study, we did not perform inferential statistics to test differences in impact between groups. Lastly, due to the cross-sectional design of the study, we do not know if the differential impacts of the COVID-19 pandemic assessed in our study represent a persistence of pre-existing health inequalities, their exacerbation, or a more complex evolution. Future studies are therefore needed to assess the longitudinal evolution of these health inequalities.

In conclusion**,** not all population groups that are vulnerable to SARS-CoV-2 infection, as well as COVID-19 related morbidity and mortality, are also susceptible to the impacts of COVID-19 across multiple other life domains. Specifically, older populations and men seem to be less vulnerable to negative changes in income status, lifestyle factors, access to non-COVID-19 health care and mental wellbeing as opposed to women and younger populations. Targeted interventions are needed in all groups that are more negatively impacted by COVID-19 in multiple life domains to prevent future risk of chronic diseases stemming from the COVID-19 pandemic.

## Data Availability

The HELIUS data are owned by the Amsterdam University Medical Centers, location AMC in Amsterdam, Netherlands. Any researcher can request the data by submitting a proposal to the HELIUS Executive Board as outlined at http://www.heliusstudy.nl/en/researchers/collaboration, by email: heliuscoordinator@amsterdamumc.nl. The HELIUS Executive Board will check proposals for compatibility with the general objectives, ethical approvals and informed consent forms of the HELIUS study. There are no other restrictions to obtaining the data and all data requests will be processed in the same manner.
