# Supplementary material for: COVID-19 Impacts Across Multiple Life Domains of Vulnerable Socio-Demographic Groups Including Migrants: A Descriptive Cross-Sectional Study
Source: Int J Public Health. 2022 May 11;67:1604665. doi: 10.3389/ijph.2022.1604665 (PMC9131879; doi:10.3389/ijph.2022.1604665)
Supplement: Supplementary file 1 [file DataSheet1.PDF]

Appendix 1. HELIUS questionnaire about the corona virus

We are very grateful for your help. On behalf of the entire HELIUS study and the Amsterdam municipality, we sincerely thank you for filling in the questionnaire!

Questionnaire 1

These questions are about the likelihood of you catching the corona virus. Please also answer these questions if you have previously been infected with the corona virus.

1. In your opinion, how likely is it that you will be infected by the corona virus in the next few months?

O Very unlikely
O Unlikely
O Neutral
O Likely
O Very likely

2. How bad would it be for you if you catch the corona virus?

O Not bad at all
O Not bad
O Neutral
O Bad
O Extremely bad

3. Suppose you did get infected by the corona virus. How likely is it, do you think, that you would infect other people?

O Very unlikely
O Unlikely
O Neutral
O Likely
O Very likely

4. How bad would you feel if you infected someone else with the corona virus?

O Not bad at all
O Not bad
O Neutral
O Bad
O Extremely bad

[page break]

5. Please indicate whether you agree or disagree with the following statements.

a. I am already immune to (protected against) the corona virus.
O Disagree completely O Disagree O Neutral O Agree O Agree completely

b. The corona virus is not very contagious.
O Disagree completely O Disagree O Neutral O Agree O Agree completely

c. I won't catch the corona virus because I never catch the flu.
O Disagree completely O Disagree O Neutral O Agree O Agree completely

d. Infections are good because they build up herd immunity.
O Disagree completely O Disagree O Neutral O Agree O Agree completely

e. I won't get very bad symptoms if I become infected with the corona virus.
O Disagree completely O Disagree O Neutral O Agree O Agree completely

f. There are enough medical healthcare facilities to take care of everyone who becomes seriously ill due to the corona virus.
O Disagree completely O Disagree O Neutral O Agree O Agree completely

g. Fasting protects you against catching the corona virus.
O Disagree completely O Disagree O Neutral O Agree O Agree completely

[page break]

6. For each of the following government measures, imagine that everyone would stick to them closely. In your opinion, how effective would each measure be in preventing the spread of the corona virus?

a. Wash your hands for 20 seconds with soap and water.
O Doesn't help O Hardly helps O Helps a bit O Helps a lot O Helps a huge amount

b. Cough and sneeze into your elbow.
O Doesn't help O Hardly helps O Helps a bit O Helps a lot O Helps a huge amount

c. Use a paper tissue to blow your nose, throw it in a bin afterwards and then wash your hands.
O Doesn't help O Hardly helps O Helps a bit O Helps a lot O Helps a huge amount

d. Don't shake hands.
O Doesn't help O Hardly helps O Helps a bit O Helps a lot O Helps a huge amount

e. Stay 1.5 metres (2 arm lengths) away from other people.
O Doesn't help O Hardly helps O Helps a bit O Helps a lot O Helps a huge amount

f. Work from home as much as possible.
O Doesn't help O Hardly helps O Helps a bit O Helps a lot O Helps a huge amount

g. If you have symptoms of a cold: stay home, don't go out shopping and don't receive any visitors.
O Doesn't help O Hardly helps O Helps a bit O Helps a lot O Helps a huge amount

h. Do not visit people whose health is already at risk.
O Doesn't help O Hardly helps O Helps a bit O Helps a lot O Helps a huge amount

i. Wear a face mask on public transport.
O Doesn't help O Hardly helps O Helps a bit O Helps a lot O Helps a huge amount

[page break]
7. Do you think there are other ways to prevent the spread of the corona virus?

……….

[page break]

8. How difficult or easy is it for you…

a. …to regularly wash your hands for 20 seconds with soap and water?
O very difficult O difficult O neutral O easy O very easy O not applicable

b. …to always cough or sneeze into your elbow (instead of into your hand or in the air)?
O very difficult O difficult O neutral O easy O very easy O not applicable

c. …to always use a paper tissue to wipe or blow your nose (instead of your sleeve or your hand, or a cotton handkerchief)?
O very difficult O difficult O neutral O easy O very easy O not applicable

d. …to stay at home as much as possible?
O very difficult O difficult O neutral O easy O very easy O not applicable

e. …to always stay 1.5 metres away from other people (except within your family/household)?
O very difficult O difficult O neutral O easy O very easy O not applicable

f. …to not visit people whose health is already at risk?
O very difficult O difficult O neutral O easy O very easy O not applicable

g. …to not shake hands?
O very difficult O difficult O neutral O easy O very easy O not applicable

h. …to wear a face mask on public transport?
O very difficult O difficult O neutral O easy O very easy O not applicable

[page break]

9. At the moment, preventing myself from becoming infected with the corona virus is...

O very difficult
O difficult
O neutral
O easy
O very easy

We are going to randomly draw ten participants who will receive a 25 euro gift card. Please fill in your email address below if you would like to participate in this draw. (If an error message appears for this question, please check if there is a space at the end of your answer. If there is a space, please delete it.)

……….

If you have any other comments that might be important for us in processing your answers, please write them here below.

……….

This is the end of the questionnaire. Our sincerest thanks for participating! Click on *Send* to complete the questionnaire.

Questionnaire 2

The following is a series of statements about the corona virus. Each statement has two extremes. For example, the corona virus feels very close or very far away. Click on the box that best describes the way you feel.

1. The corona virus…

a. Feels very close [*] [*] [*] [*] [*] feels very far away

b. Spreads very quickly [*] [*] [*] [*] [*] spreads very slowly

c. Is something I think of all the time [*] [*] [*] [*] [*] is something I hardly ever think of

d. Frightens me a lot [*] [*] [*] [*] [*] doesn't frighten me at all

e. Worries me a great deal [*] [*] [*] [*] [*] doesn't worry me at all

f. Makes me feel helpless [*] [*] [*] [*] [*] doesn't make me feel helpless

g. Causes me great stress [*] [*] [*] [*] [*] doesn't cause me any stress at all

h. Is being exaggerated by the media [*] [*] [*] [*] [*] is not being exaggerated by the media at all

[page break]

The following questions are about news and information concerning the corona virus.

2. Which sources were important for you during the past week for getting news and information about the corona virus? Multiple answers possible.

- □ I didn't follow news or information about the corona virus during the past week
- □ Dutch government website or RIVM website
- □ My local GGD's website or website of my local municipality
- □ Family doctor / GP
- □ Medical websites such as thuisarts.nl
- □ National newspapers
- □ Regional and local media
- □ News/newspapers from a country other than the Netherlands → proceed to question 2b
- □ Online news sites or apps, such as nos.nl or nu.nl
- □ Dutch government's press conference on TV
- □ Social media, such as Facebook, Twitter, or Instagram
- □ People around me, such as neighbours, colleagues, or family
- □ News broadcasts on Dutch public or commercial channels, such as NOS Journaal or RTL Nieuws
- □ Dutch current affairs programmes, such as Nieuwsuur or EenVandaag
- □ Dutch talk shows, such as Op1, Jinek, or M
- □ Radio
- □ Religious leader/preacher in mosque, church, etc.
- □ Communication from my employer
- □ Other, namely…

→*skip question if 'News/newspapers from a country other than the Netherlands' was not selected in question 2*

2b. Do you follow the news from other countries besides the Netherlands? If so, which country/countries?

- o Suriname
- o Turkey
- o Morocco

- o Ghana
- o Other, namely → proceed to question 2c

→skip question if 'Other, namely' was not selected in question 2b
2c. You indicated that you follow news from other countries than previously stated. From which country/countries?

…..

[page break]

3. In the past 7 days (1 week), have you spoken with other people about the Dutch government's approach to the corona virus? By 'approach' we mean: the measures the government takes to keep the virus under control.

O No > PROCEED TO QUESTION 5
O Yes, once or twice
O Yes, almost every day
O Yes, every day
O Yes, multiple times every day

4. Were these conversations about the Dutch government's approach generally negative or positive?

O Very negative
O Negative
O Neutral
O Positive
O Very positive

5. How much confidence do you have in the way the Dutch government is trying to keep the corona virus under control?

O No confidence at all
O No confidence
O Neutral
O Lots of confidence
O A huge amount of confidence

6. The following is a statement with two extremes. Click on the box that best describes your opinion of the way the Dutch government is trying to keep the corona virus under control.

The Dutch government is doing a worse job than other countries [*] [*] [*] [*] [*] The Dutch government is doing a better job than other countries

[page break]

The following questions are about what you have been doing for the **past 7 days (1 week)**.

7. Compared to the period before the corona measures, I exercise and participate in sports…

O Much less
O Less
O The same amount
O More
O Much more

8. Compared to the period before the corona measures, I eat…

O A much less healthy diet
O A less healthy diet
O The same
O A more healthy diet
O A much healthier diet

9. Compared to the period before the corona measures, I drink…

O Much less alcohol
O Less alcohol
O The same amount of alcohol
O More alcohol
O Much more alcohol
O Not applicable, I don't drink alcohol

10**. Compared to the period before the corona measures, I smoke…**

O Much less tobacco
O Less tobacco
O The same amount of tobacco
O More tobacco
O Much more tobacco
O Not applicable, I don't smoke

We are going to randomly draw ten participants who will receive a 25 euro gift card. Please fill in your email address below if you would like to participate in this draw. (If an error message appears for this question, please check if there is a space at the end of your answer. If there is a space, please delete it.)

……….

If you have any other comments that might be important for us in processing your answers, please write them here below.

……….

This is the end of the questionnaire. Our sincerest thanks for participating! Click on *Send* to complete the questionnaire.

Questionnaire 3

The corona measures may have little impact on some people's lives. Other people might be more affected by them. The following questions are about the effects of these measures on your life, and how you feel.

1. What is your current work situation?

Multiple answers possible.
O Paid work in a permanent job → PROCEED TO QUESTION 3
O Self-employed (ZZP)/ run my own company → PROCEED TO QUESTION 3
O Volunteer work → PROCEED TO QUESTION 3
O Retired (or early retirement) → PROCEED TO QUESTION 4
O Unemployed or jobseeker → PROCEED TO QUESTION 2, then proceed to question 4
O Unfit for work → PROCEED TO QUESTION 4
O On welfare benefits → PROCEED TO QUESTION 2, then proceed to question 4
O Homemaker (male or female) → PROCEED TO QUESTION 4
O Attending school / college → PROCEED TO QUESTION 4
O None of the above → PROCEED TO QUESTION 4

2. In answer to the previous question you indicated that you are unemployed or seeking work, and/or that you are receiving welfare benefits. Is this a consequence of the corona crisis?
O Yes
O No

3. What is the current status of your work situation (including volunteer work)? Multiple answers possible.
□ My work is continuing as usual
□ My work has stopped
□ I am no longer working
□ I am currently working less
□ I am currently doing different work than I normally do
□ I am (mostly) working from home now
□ Other, namely…

[page break]

4. In the past year, have you had trouble getting by on your family income?
_ No, no trouble at all → proceed to question 6
_ No, no trouble, but I have to be careful about how much I spend → proceed to question 6
_ Yes, some trouble → proceed to question 5
_ Yes, a lot of trouble → proceed to question 5

5. Are you having more trouble getting by on your family income now than before the corona crisis?
Yes
No
I don't know

6. Compared to the period before the corona measures, I feel…

O Much less stressed
O Less stressed
O The same
O More stressed
O A lot more stressed
O Not applicable, I never feel stressed

7. Compared to the period before the corona measures, I am having…

O Much less trouble sleeping
O Less trouble sleeping
O The same
O More trouble sleeping
O Much more trouble sleeping
O Not applicable, I never have trouble sleeping

[page break]

The following questions are about your social contacts with other people in this period.

8. Please indicate for each of the following statements to what extent they apply to you, specifically in relation to the past 7 days (1 week).

a. I experience emptiness around me.	O Yes	O Somewhat	O No
b. There are enough people I can fall back on in case of trouble.	O Yes	O Somewhat	O No
c. I have lots of people I can rely on.	O Yes	O Somewhat	O No
d. I miss having people around me.	O Yes	O Somewhat	O No
e. There are enough people with whom I feel closely connected.	O Yes	O Somewhat	O No
f. I often feel abandoned.	O Yes	O Somewhat	O No

[page break]

9. Compared to the period before the corona measures, I feel…
O Much less lonely
O Less lonely
O The same
O More lonely
O Much more lonely
O Not applicable, I never feel lonely

[page break]

10. Looking back over <u>the past 7 days (1 week)</u>, how much do you disagree or agree with the following statements?

a. I feel supported by other people.
O Disagree completely
O Disagree
O Neutral
O Agree
O Agree completely

b. I feel I have good contact with my family, friends and acquaintances.
O Disagree completely
O Disagree
O Neutral
O Agree
O Agree completely

c. I currently experience digital/phone contact as positive.
O Disagree completely
O Disagree
O Neutral
O Agree
O Agree completely

d. All things considered, I feel positive about the quality of my social contacts.
O Disagree completely
O Disagree
O Neutral
O Agree
O Agree completely

e. Compared to the period before the corona measures, the quality of my social contacts is better.
O Disagree completely
O Disagree
O Neutral
O Agree
O Agree completely

[page break]

11. In the past two weeks, how often have you suffered from the following problems?
Array using the following answers: never; on a few days; on more than half the days; almost every day
a. Little urge or enthusiasm to do anything.
b. Sombreness, dejection, pessimism.
c. Trouble falling asleep or staying asleep, or sleeping too much.
d. Fatigue, lack of energy.
e. Lack of appetite or eating too much.
f. Are you dissatisfied with yourself, or do you feel you are a failure, or that you are inadequate for yourself or your loved ones?
g. Do you have trouble concentrating, for example concentrating on reading the newspaper or watching TV?
h. Do you move or speak so slowly that other people might notice?
i. Do you feel so nervous that you are more restless or fidgety than you normally are?
j. Have you thought you would be better off dead, or have you thought about harming yourself?

We are going to randomly draw ten participants who will receive a 25 euro gift card. Please fill in your email address below if you would like to participate in this draw. (If an error message appears for this question, please check if there is a space at the end of your answer. If there is a space, please delete it.)

……….

If you have any other comments that might be important for us in processing your answers, please write them here below.

……….

This is the end of the questionnaire. Our sincerest thanks for participating! Click on *Send* to complete the questionnaire.

Questionnaire 4

The Dutch government is currently recommending a series of measures to combat the spread of the corona virus. Please indicate for each of these measures whether you currently recognise its importance and therefore support the measure.

1. To what extent do you support the measures that are in place now?

a. Wash your hands for 20 seconds with soap and water.
O Strongly do not support O Do not support O Neutral O Support O Strongly support O No opinion

b. Cough and sneeze into your elbow.
O Strongly do not support O Do not support O Neutral O Support O Strongly support O No opinion

c. Use a paper tissue to blow your nose, throw it in a bin afterwards and then wash your hands.
O Strongly do not support O Do not support O Neutral O Support O Strongly support O No opinion

d. Don't shake hands.
O Strongly do not support O Do not support O Neutral O Support O Strongly support O No opinion

e. Stay 1.5 metres (2 arm lengths) away from other people.
O Strongly do not support O Do not support O Neutral O Support O Strongly support O No opinion

f. Work from home as much as possible.
O Strongly do not support O Do not support O Neutral O Support O Strongly support O No opinion

g. If you have symptoms of a cold: stay home, don't go out shopping and don't receive any visitors.
O Strongly do not support O Do not support O Neutral O Support O Strongly support O No opinion

h. Do not visit people whose health is already at risk.
O Strongly do not support O Do not support O Neutral O Support O Strongly support O No opinion

i. Wear a face mask on public transport.
O Strongly do not support O Do not support O Neutral O Support O Strongly support O No opinion

[page break]

2. To what extent would you support the measures if they would last for another 6 months?

a. Wash your hands for 20 seconds with soap and water.
O Strongly do not support O Do not support O Neutral O Support O Strongly support O No opinion

b. Cough and sneeze into your elbow.
O Strongly do not support O Do not support O Neutral O Support O Strongly support O No opinion

c. Use a paper tissue to blow your nose, throw it in a bin afterwards and then wash your hands.
O Strongly do not support O Do not support O Neutral O Support O Strongly support O No opinion

d. Don't shake hands.
O Strongly do not support O Do not support O Neutral O Support O Strongly support O No opinion

e. Stay 1.5 metres (2 arm lengths) away from other people.
O Strongly do not support O Do not support O Neutral O Support O Strongly support O No opinion

f. Work from home as much as possible.

O Strongly do not support O Do not support O Neutral O Support O Strongly support O No opinion

g. If you have symptoms of a cold: stay home, don't go out shopping and don't receive any visitors.
O Strongly do not support O Do not support O Neutral O Support O Strongly support O No opinion

h. Do not visit people whose health is already at risk.
O Strongly do not support O Do not support O Neutral O Support O Strongly support O No opinion

i. Wear a face mask on public transport.
O Strongly do not support O Do not support O Neutral O Support O Strongly support O No opinion

[page break]

The government advises citizens to stick to the rules in order to combat the corona virus. Many people do their best to comply with this advice. Sometimes this can be difficult. For example, if you're in a hurry, if you're tired or when you're visiting people. Maybe you don't always manage to wash your hands for 20 seconds, or to stay 1.5 metres away from other people. Or perhaps you don't agree with the measures, so you choose not to comply with them.

We would like to get a picture of what you did and didn't do in the past 7 days (1 week).
If you can't recall exactly, please try to estimate as precisely as possible.

3. In the past 7 days (1 week), how often did you wash your hands with soap and water?

a. before you left the house
O never O rarely O sometimes O regularly O often O usually O always O not applicable

b. when you came back home
O never O rarely O sometimes O regularly O often O usually O always O not applicable

c. when you visited someone in their home
O never O rarely O sometimes O regularly O often O usually O always O not applicable

d. before you ate (breakfast, lunch, evening meal, snacks)
O never O rarely O sometimes O regularly O often O usually O always O not applicable

e. after going to the toilet
O never O rarely O sometimes O regularly O often O usually O always O not applicable

f. after blowing your nose
O never O rarely O sometimes O regularly O often O usually O always O not applicable

[page break]

The following recommendations may seem simple, but for some people they are difficult because these are things people do automatically, without thinking.

4. If you have a cold or a runny nose, the recommendation is to wipe or blow your nose with a paper tissue.

In the <u>past 7 days (1 week)</u>, how often did you use a paper tissue to wipe or blow your nose, instead of your sleeve, your hand, or a handkerchief made of cotton/other fabric?

O Never
O Rarely
O Sometimes
O Regularly
O Often
O Usually
O Always
O Not applicable: I did not have a cold or a runny nose

5. When you sneeze or cough, the recommendation is to sneeze or cough into your elbow.
In the <u>past 7 days (1 week),</u> how often did you cough or sneeze into your elbow instead of coughing or sneezing into your hand, or not covering your mouth at all?

O Never
O Rarely
O Sometimes
O Regularly
O Often
O Usually
O Always
O Not applicable: I didn't cough/sneeze

[page break]

6.

The following statements are about healthcare services. Please indicate to what extent you agree with the statements.

	agree completely	agree	neither agree nor disagree	disagree	disagree completely	not applicable
I am reluctant to go to the doctor for fear of catching the corona virus.						I
I am reluctant to allow caregivers (*e.g., district nurse*) into my home for fear of catching the corona virus.						I
I am currently being denied medical treatment due to the corona virus.						
I currently receive less professional care than before the corona crisis						

(e.g., daytime activities, domestic help, or district nurse).

We are going to randomly draw ten participants who will receive a 25 euro gift card. Please fill in your email address below if you would like to participate in this draw. (If an error message appears for this question, please check if there is a space at the end of your answer. If there is a space, please delete it.)

……….

If you have any other comments that might be important for us in processing your answers, please write them here below.

……….

This is the end of the questionnaire. Our sincerest thanks for participating! Click on *Send* to complete the questionnaire.

Appendix 2. Baseline characteristics of responders compared to non-responders and total Helius population.

Characteristic	All HELIUS participants (N= 24,789)[1]	Online questionnaire Participants (n=4450)	Online questionnaire Non-responders (n=8581)	P-value[2]	P-value[3]
	n (%)	n (%)	n (%)		
**Migration background**				<0.001	1
Dutch origin	4671 (18.8%)	1924 (43.2%)	1377 (16.0%)		
South-Asian Surinamese origin	3368 (13.6%)	610 (13.7%)	1114 (13.0%)		
African Surinamese origin	4458 (18.0%)	735 (16.5%)	1642 (19.1%)		
Other/unknown Surinamese origin	803 (3.2%)	140 (3.1%)	288 (3.4%)		
Ghanaian origin	2735 (11.0%)	112 (2.5%)	655 (7.6%)		
Turkish origin	4200 (16.9%)	407 (9.1%)	1469 (17.1%)		
Moroccan origin	4502 (18.2%)	506 (11.4%)	2023 (23.6%)		
Other/unknown/missing	51 (0.2%)	16 (0.4%)	13 (0.2%)		
**Sex**				1	1
Male	10554 (42.6%)	1931 (43.4%)	3804 (44.3%)		
Female	14234 (57.4%)	2519 (56.6%)	4777 (55.7%)		
**Age in years on 1 January 2020**					
Median [IQR]	52 [41-61]	54 [43-62]	49[38-58]	<0.001	<0.001
**Age categories (years)[1]**				0.552	0.078
<40	6902(24.7%)	815(18.6%)	2422 (29.7%)		
40-65	14767(59.6%)	2708(61.8%)	4854 (59.5%)		
>65	3119(12.6%)	860 (19.6%)	884 (10.8%)		
**Migration generation**				1	0.329
N.A. (Dutch group)	4671 (18.8%)	1924 (43.2%)	1377 (16.0%)		
1st	10737 (43.4%)	1937 (43.5%)	5076 (59.2%)		
2nd	4709 (19.0%)	589 (13.2%)	2128 (24.8%)		
Missing	4671 (18.8%)	0 (0.0%)	0 (0.0%)		
**Educational level**				<0.001	0.015
No school/elementary school	4188 (16.9%)	205 (4.6%)	1056 (12.3%)		
Lower secondary school	6332 (25.5%)	864 (19.4%)	2162 (25.2%)		
Intermediary secondary school	7071 (28.5%)	1292 (29.0%)	2833 (33.0%)		
Higher vocational/university	6130 (24.7%)	2021 (45.4%)	2178 (25.4%)		
Missing	1067 (4.3%)	68 (1.5%)	352 (4.1%)		
**Professional level**				<0.008	0.022
Elementary occupations	3173 (12.8%)	184 (4.1%)	790 (9.2%)		
Lower occupations	6196 (25.0%)	816 (18.3%)	2225 (25.9%)		
Intermediary occupations	5373 (21.7%)	1206 (27.1%)	2148 (25.0%)		
Higher occupations	3911 (15.8%)	1280 (28.8%)	1447 (16.9%)		
Scientific occupations	1421 (5.7%)	581 (13.1%)	424 (4.9%)		
Missing	4714 (19.0%)	383 (8.6%)	1547 (18.0%)		
**Difficulty with Dutch language**				0.015	0.114
N.A. (Dutch group)	4671 (18.8%)	1924 (43.2%)	1377 (16.0%)		
No	11412(46.0%)	1883 (42.3%)	4567 (53.2%)		

Yes	7684 (31.0%)	566 (12.7%)	2316 (27.0%)		
Missing	5692 (23.0%)	77 (1.73%)	321 (3.8%)		
**Health literacy (SBSQ)**				0.031	0.043
Adequate	20313 (81.9%)	4258 (95.7%)	7443 (86.7%)	0.006	0.046
Low	3517 (14.2%)	132 (3.0%)	827 (9.6%)		
Missing	958 (3.9%)	60 (1.3%)	311 (3.6%)		

[1] P for difference in characteristic between Online questionnaire participants and total HELIUS population. P-values obtained by perfoming (chi-square test on proportions (percentages) of a characteristic between groups (categorical variables), or Kruskal-wallis test for the median age.

[2] P for difference in characteristic between Online questionnaire participants and online questionnaire non-responders. P-values obtained by perfoming (chi-square test on proportions (percentages) of a characteristic between groups (categorical variables), or Kruskal-wallis test for the median age.

SBSQ= set of brief screening questions

Appendix 3. Comparison of baseline characteristics across questionnaire sections one to four.

Characteristic	Questionnaire Part 1 (N= 1084)	Questionnaire Part 2 (n=1123)	Questionnaire Part 3 (n=1138)	Questionnaire Part 4 (n=1105)	P-value[1]
	n (%)	n (%)	n (%)	n (%)	
**Migration background**					0.768
Dutch origin	459 (42.3%)	493(43.9%)	511(44.9%)	461(41.7%)	
South-Asian Surinamese origin	158(14.6%)	156(13.9%)	158(13.9%)	138(12.5%)	
African Surinamese origin	186(17.2%)	184(16.4%)	170(14.9%)	195(17.6%)	
Other Surinamese origin	37(3.4%)	37(3.3%)	34(3.0%)	32(2.9%)	
Ghanaian origin	24(2.2%)	29(2.6%)	28(2.5%)	31(2.8%)	
Turkish origin	109(10.1%)	103(9.2%)	90(7.9%)	105(9.5%)	
Moroccan origin	108(10.0%)	117(10.4%)	142(12.5%)	139(12.6%)	
Other/unknown/missing	3(0.3%)	4(0.4%)	5(0.4%)	4(0.4%)	
**Gender**					0.689
Male	472(43.5%)	487(43.4%)	479(42.1%)	493(44.6%)	
Female	612(56.5%)	636(56.6%)	659(57.9%)	612(55.4%)	
**Age in years on 1 January 2020**					
Median [IQR]	55[43-63]	54[43-62]	55[44-63]	55[45-64]	0.116
**Age categories (years)**					0.199
<40	192(17.9%)	226(20.4%)	210(18.7%)	187(17.3%)	
40-65	675(63.0%)	682(61.7%)	692(61.7%)	659(60.8%)	
>65	205(19.1%)	198(17.9%)	219(19.5%)	238(22.0%)	
**Migration** generation					0.472
1st	469(43.3%)	477(42.5%)	487(42.8%)	504(45.6%)	
2nd	156(14.4%)	153(13.6%)	140(12.3%)	140(12.7%)	
Missing/Not applicable (Dutch)	459(42.3%)	493(43.9%)	511(44.9%)	461(41.7%)	
**Educational level**					0.189
No school/elementary school	53(4.9%)	40(3.6%)	51(4.5%)	61(5.5%)	
Lower secondary school	213(19.6%)	204(18.2%)	217(19.1%)	230(20.8%)	
Intermediary secondary school	329(30.4%)	332(29.6%)	340(29.9%)	291(26.3%)	
Higher vocational/university	473(43.6%)	531(47.3%)	514(45.2%)	503(45.5%)	
Missing	16(1.5%)	16(1.4%)	16(1.4%)	20(1.8%)	
**Professional level**					0.735
Elementary occupations	42(3.9%)	47(4.2%)	51(4.5%)	44(4.0%)	
Lower occupations	212(19.6%)	183(16.3%)	206(18.1%)	215(19.5%)	
Intermediary occupations	291(26.8%)	321(28.6%)	307(27.0%)	287(26.0%)	
Higher occupations	325(30.0%)	330(29.4%)	309(27.2%)	316(28.6%)	
Scientific occupations	136(12.5%)	148(13.2%)	158(13.9%)	139(12.6%)	
Missing	78(7.2%)	94(8.4%)	107(9.4%)	104(9.4%)	
**Difficulty with Dutch language**					0.574
No	476(43.9%)	473(42.1%)	456(40.1%)	478(43.3%)	
Yes	133(12.3%)	136(12.1%)	152(13.4%)	145(13.1%)	
Missing/Not applicable (Dutch)	475(43.8%)	514(45.8%)	530(46.6%)	482(43.6%)	
**Health literacy**					0.101

Adequate	1031(95.1%)	1081(96.3%)	1090(95.8%)	1056(95.6%)	0.483
Low	36(3.3%)	26(2.3%)	34(3.0%)	36(3.3%)	
Missing	17(1.6%)	16(1.4%)	14(1.2%)	13(1.2%)	

[1] P-value for differences in baseline characteristics between the ethnic groups. P-value obtained via chi-square test for categorical variables, or Kruskal wallis test for median age.

SBSQ= set of brief screening questions

Appendix 4. Changes in multiple life domains due to the COVID-19 by migration background.

	Within group N	Crude	Age and sex adjusted
		**% (95% CI)[1]**	**% (95% CI)[2]**
**A. Changes in job status and income**			
Of those that are jobless, the cause is the coronavirus pandemic ... (yes)?[3]			
African Surinamese origin	13	23(8-47)	14(7-21)
Dutch origin	21	24(10-42)	27(18-35)
Ghanaian origin	4	25(0-60)	16(3-29)
Moroccan origin	19	26(11-46)	26(15-36)
South-Asian Surinamese origin	10	30(10-60)	28(18-38)
Turkish origin	11	18(9-44)	15(6-25)
Of those with trouble getting by family income, the cause is the coronavirus pandemic... (yes)?[4]			
African Surinamese origin	40	70(58-87)	49(42-57)
Dutch origin	67	76(66-86)	70(64-75)
Ghanaian origin	4	67(33-100)	29(17-41)
Moroccan origin	41	74(63-89)	62(54-69)
South-Asian Surinamese origin	31	66(52-84)	63(54-72)
Turkish origin	36	66(52-84)	50(42-58)
**B. Changes to less healthy behavior**			
Compared to the period before the corona measures, I exercise and participate in sports... (less + much less)?			
African Surinamese origin	184	49(42-57)	49(46-53)
Dutch origin	493	35(30-40)	34(32-36)
Ghanaian origin	29	52(36-74)	48(39-58)
Moroccan origin	117	48(39-58)	45(40-50)
South-Asian Surinamese origin	156	56(48-64)	55(51-59)
Turkish origin	103	39(30-50)	30(26-34)
Compared to the period before the corona measures, I eat... (less healthy + much less healthy)?			
African Surinamese origin	184	10(4-17)	12(9-14)
Dutch origin	493	6(3-10)	6(5-7)
Ghanaian origin	29	12(0-35)	14(7-20)
Moroccan origin	117	8(1-17)	5(3-6)
South-Asian Surinamese origin	156	16(8-23)	14(12-17)
Turkish origin	103	10(2-18)	8(5-10)
Compared to the period before the corona measures, I drink... (much alcohol + much more alcohol)?[5]			
African Surinamese origin	79	6(0-18)	7(4-10)
Dutch origin	385	12(8-17)	13(11-15)
Ghanaian origin	11	9(0-45)	5(2-9)
Moroccan origin	8	0(0-41)	0(0-0)
South-Asian Surinamese origin	60	18(7-32)	18(13-22)
Turkish origin	30	17(0-34)	15(9-20)
Compared to the period before the corona measures, I smoke... (much tobacco + much more tobacco)?[5]			
African Surinamese origin	79	6(0-18)	7(4-10)
Dutch origin	385	12(8-17)	13(11-15)

Ghanaian origin	11	9(0-45)	5(2-9)
Moroccan origin	8	0(0-41)	0(0-0)
South-Asian Surinamese origin	60	18(7-32)	18(13-22)
Turkish origin	30	17(0-34)	15(9-20)
**C. Changes in mental health factors**			
Compared to the period before the corona measures, I am having sleep... (more trouble sleeping + much more trouble sleeping)?[5]			
African Surinamese origin	143	23(16-31)	30(27-34)
Dutch origin	461	18(14-21)	18(16-20)
Ghanaian origin	23	13(0-31)	10(5-16)
Moroccan origin	131	34(25-42)	41(36-45)
South-Asian Surinamese origin	140	32(24-41)	32(28-36)
Turkish origin	82	40(30-52)	37(32-42)
Compared to the period before the corona measures, I am having stress... (more stressed + much more stressed)?[5]			
African Surinamese origin	150	48(40-57)	48(44-53)
Dutch origin	490	36(31-41)	36(34-38)
Ghanaian origin	26	27(12-50)	19(13-26)
Moroccan origin	138	56(48-65)	61(57-66)
South-Asian Surinamese origin	145	43(34-51)	42(38-46)
Turkish origin	89	60(51-71)	60(54-66)
Compared to the period before the corona measures, I am having lonely... (more lonely + much more lonely)?[5]			
African Surinamese origin	136	25(18-33)	23(19-26)
Dutch origin	428	31(27-36)	31(28-33)
Ghanaian origin	25	16(0-36)	15(9-21)
Moroccan origin	129	33(24-41)	29(26-33)
South-Asian Surinamese origin	132	31(23-39)	30(26-34)
Turkish origin	85	45(34-56)	50(45-56)
**D. Changes in access to non-COVID care**			
I am reluctant to go to the doctor for fear of catching the corona virus.... (agree + agree completely)?[5]			
African Surinamese origin	193	19(11-26)	17(14-19)
Dutch origin	457	9(4-14)	9(7-10)
Ghanaian origin	30	30(13-49)	27(21-34)
Moroccan origin	131	25(17-35)	30(26-33)
South-Asian Surinamese origin	134	34(25-43)	30(27-34)
Turkish origin	99	41(31-52)	46(41-51)
I am reluctant to allow caregivers into my home for fear of catching the corona virus.... (agree + agree completely)?[5]			
African Surinamese origin	193	23(16-31)	24(21-28)
Dutch origin	457	11(6-16)	11(9-12)
Ghanaian origin	30	33(17-52)	27(21-34)
Moroccan origin	131	32(24-42)	36(32-39)
South-Asian Surinamese origin	134	34(26-44)	32(28-36)
Turkish origin	99	35(25-46)	43(38-48)
I am currently being denied medical treatment due to the corona virus… (agree + agree completely)?[5]			
African Surinamese origin	118	16(7-25)	14(11-17)
Dutch origin	223	16(9-23)	17(14-19)

Ghanaian origin	18	22(6-49)	14(9-20)
Moroccan origin	66	39(27-52)	41(33-49)
South-Asian Surinamese origin	86	26(15-37)	24(19-28)
Turkish origin	51	29(16-43)	29(22-36)
I currently receive less professional care than before the corona crisis… (agree + agree completely)?[5]			
African Surinamese origin	61	11(0-25)	11(7-15)
Dutch origin	119	16(7-25)	16(13-20)
Ghanaian origin	14	21(0-43)	25(15-34)
Moroccan origin	44	43(30-59)	44(36-53)
South-Asian Surinamese origin	48	29(17-45)	30(24-37)
Turkish origin	37	32(19-51)	31(23-38)

[1] crude proportion (95% confidence intervals).

[2] proportion (95% confidence intervals) adjusted for age and sex. Proportions obtained using adj. prop package in R. Adjustments were done according to the structure of the whole HELIUS COVID-19 sub-sample.

[3] total number of participants that reported as jobless (unemployed or job seeker) was **84**
adjusted proportions in age category were only adjusted for sex.

[4] total number of participants that reported as trouble with family income was **219**

[5] calculated only in those that apply

Within group totals do not always add to expected totals due to missings.

Appendix 5. Changes in multiple life domains due to the COVID-19 by age groups.

	Within group N	Crude % (95% CI)[1]	Sex adjusted % (95% CI)[2]
**A. Changes in job status and income**			
Of those that are jobless, the cause is the coronavirus pandemic ... (yes)?[3]			
<40 years	13	23(8-47)	25(13-37)
40-65 years	60	25(15-36)	25(19-30)
>65 years	3	33(0-69)	23(7-39)
Of those with trouble getting by family income, the cause is the coronavirus pandemic... (yes)?[4]			
<40 years	35	72(59-89)	67(59-75)
40-65 years	150	73(66-81)	62(58-66)
>65 years	29	59(45-79)	61(52-71)
**B. Changes to less healthy behavior**			
Compared to the period before the corona measures, I exercise and participate in sports... (less + much less)?			
<40 years	219	45(38-52)	45(41-48)
40-65 years	655	42(38-46)	42(40-44)
>65 years	191	43(35-50)	44(41-48)
Compared to the period before the corona measures, I eat... (less healthy + much less healthy)?			
<40 years	219	14(8-21)	15(12-17)
40-65 years	655	8(5-12)	8(7-9)
>65 years	191	5(0-10)	5(4-7)
Compared to the period before the corona measures, I drink... (much alcohol + much more alcohol)?[5]			
<40 years	107	16(7-26)	16(12-20)
40-65 years	333	14(9-19)	14(12-16)
>65 years	130	4(0-11)	4(2-6)
Compared to the period before the corona measures, I smoke... (much tobacco + much more tobacco)?[5]			
<40 years	39	23(8-39)	23(16-30)
40-65 years	108	18(9-27)	18(14-21)
>65 years	27	15(0-31)	16(9-23)
**C. Changes in mental health factors**			
Compared to the period before the corona measures, I am having sleep... (more trouble sleeping + much more trouble sleeping)?[5]			
<40 years	178	29(22-36)	29(26-33)
40-65 years	603	25(22-29)	25(23-27)
>65 years	186	17(11-22)	17(14-19)
Compared to the period before the corona measures, I am having stress... (more stressed + much more stressed)?[5]			
<40 years	202	46(39-53)	45(42-49)
40-65 years	634	45(41-49)	45(43-47)
>65 years	186	32(25-40)	32(29-36)
Compared to the period before the corona measures, I am having lonely... (more lonely + much more lonely)?[5]			
<40 years	179	34(27-41)	33(29-36)
40-65 years	572	31(27-35)	31(29-33)
>65 years	171	29(23-37)	29(26-33)
**D. Changes in access to non-COVID care**			

I am reluctant to go to the doctor for fear of catching the corona virus.... (agree + agree completely)?[5]			
<40 years	176	20(12-28)	21(17-24)
40-65 years	620	20(16-25)	20(19-22)
>65 years	227	15(9-22)	16(13-18)
I am reluctant to allow caregivers into my home for fear of catching the corona virus.... (agree + agree completely)?[5]			
<40 years	176	22(14-30)	21(18-25)
40-65 years	620	23(19-27)	23(21-25)
>65 years	227	19(12-25)	18(16-21)
I am currently being denied medical treatment due to the corona virus... (agree + agree completely)?[5]			
<40 years	78	26(15-38)	28(22-33)
40-65 years	324	22(16-28)	22(19-24)
>65 years	147	19(11-28)	20(16-23)
I currently receive less professional care than before the corona crisis... (agree + agree completely)?[5]			
<40 years	52	25(12-39)	26(19-32)
40-65 years	196	26(18-33)	25(22-28)
>65 years	64	17(6-31)	17(12-23)

[1] crude proportion (95% confidence intervals).

[2] proportion (95% confidence intervals) adjusted for sex. Proportions obtained using adj. prop package in R. Adjustments were done according to the structure of the whole HELIUS COVID-19 sub-sample.

[3] total number of participants that reported as jobless (unemployed or job seeker) was **84**
adjusted proportions in age category were only adjusted for sex.

[4] total number of participants that reported as trouble with family income was **219**

[5] calculated only in those that apply

Within group totals do not always add to expected totals due to missings.

Appendix 6. Changes in multiple life domains due to the COVID-19 by sex.

	Within group N	Crude	Age adjusted
		% (95% CI)[1]	% (95% CI)[2]
**A. Changes in job status and income**			
Of those that are jobless, the cause is the coronavirus pandemic ... (yes)?[3]			
Men	36	33(19-49)	33(25-41)
Women	42	17(7-27)	17(11-23)
Of those with trouble getting by family income, the cause is the coronavirus pandemic... (yes)?[4]			
Men	108	66(56-75)	57(52-62)
Women	111	77(69-85)	68(64-73)
**B. Changes to less healthy behavior**			
Compared to the period before the corona measures, I exercise and participate in sports... (less + much less)?			
Men	471	39(34-44)	40(38-42)
Women	611	45(41-50)	46(43-48)
Compared to the period before the corona measures, I eat... (less healthy + much less healthy)?			
Men	471	8(4-12)	8(7-10)
Women	611	10(6-13)	10(8-11)
Compared to the period before the corona measures, I drink... (much alcohol + much more alcohol)?[5]			
Men	281	12(6-17)	12(10-14)
Women	292	12(7-18)	12(10-14)
Compared to the period before the corona measures, I smoke... (much tobacco + much more tobacco)?[5]			
Men	88	11(2-21)	11(8-15)
Women	92	26(16-37)	26(21-30)
**C. Changes in mental health factors**			
Compared to the period before the corona measures, I am having sleep... (more trouble sleeping + much more trouble sleeping)?[5]			
Men	420	24(20-28)	24(22-26)
Women	560	25(21-29)	25(23-27)
Compared to the period before the corona measures, I am having stress... (more stressed + much more stressed)?[5]			
Men	436	39(35-45)	39(37-42)
Women	602	46(41-50)	45(43-47)
Compared to the period before the corona measures, I am having lonely... (more lonely + much more lonely)?[5]			
Men	406	28(23-33)	28(26-30)
Women	529	34(30-38)	34(32-36)
**D. Changes in access to non-COVID care**			
I am reluctant to go to the doctor for fear of catching the corona virus.... (agree + agree completely)?[5]			
Men	460	20(15-25)	20(18-22)
Women	584	20(15-24)	19(17-21)
I am reluctant to allow caregivers into my home for fear of catching the corona virus.... (agree + agree completely)?[5]			
Men	460	21(16-26)	21(19-23)
Women	584	23(18-27)	22(20-24)
I am currently being denied medical treatment due to the corona virus... (agree + agree completely)?[5]			
Men	260	19(13-26)	20(18-23)
Women	302	24(18-29)	24(21-26)

I currently receive less professional care than before the corona crisis… (agree + agree completely)?[5]			
Men	156	20(12-29)	22(18-25)
Women	167	26(18-34)	26(22-29)

[1] crude proportion (95% confidence intervals).
[2] proportion (95% confidence intervals) adjusted for sex. Proportions obtained using adj. prop package in R. Adjustments were done according to the structure of the whole HELIUS COVID-19 sub-sample.
[3] total number of participants that reported as jobless (unemployed or job seeker) was **84**
adjusted proportions in age category were only adjusted for sex.
[4] total number of participants that reported as trouble with family income was **219**
[5] calculated only in those that apply
Within group totals do not always add to expected totals due to missings.

Appendix 7. Changes in multiple life domains due to the COVID-19 by education.
.

	Within group N	Crude	Age and sex adjusted
		% (95% CI)[1]	% (95% CI)[2]
**A. Changes in job status and income**			
Of those that are jobless, the cause is the coronavirus pandemic ... (yes)?[3]			
No school/elementary school	7	29(14-68)	22(9-36)
Lower secondary school	25	32(16-50)	27(20-34)
Intermediary secondary school	26	19(8-35)	18(11-25)
Higher vocational/university	19	16(5-33)	12(6-18)
Of those with trouble getting by family income, the cause is the coronavirus pandemic... (yes)?[4]			
No school/elementary school	21	74(58-94)	63(53-72)
Lower secondary school	64	64(53-78)	57(52-62)
Intermediary secondary school	68	71(61-83)	58(52-64)
Higher vocational/university	63	76(66-86)	71(65-76)
**B. Changes to less healthy behavior**			
Compared to the period before the corona measures, I exercise and participate in sports... (less + much less)?			
No school/elementary school	39	42(28-60)	42(36-48)
Lower secondary school	200	43(36-51)	42(38-46)
Intermediary secondary school	319	46(40-52)	47(44-50)
Higher vocational/university	520	40(36-45)	40(38-43)
Compared to the period before the corona measures, I eat... (less healthy + much less healthy)?			
No school/elementary school	39	6(0-17)	6(2-9)
Lower secondary school	200	8(2-14)	7(5-9)
Intermediary secondary school	319	11(6-16)	10(8-12)
Higher vocational/university	520	9(5-12)	9(8-10)
Compared to the period before the corona measures, I drink... (much alcohol + much more alcohol)?[5]			
No school/elementary school	9	0(0-39)	0(0-0)
Lower secondary school	74	4(0-15)	4(2-6)
Intermediary secondary school	143	16(8-25)	16(13-19)
Higher vocational/university	347	12(7-18)	13(11-15)
Compared to the period before the corona measures, I smoke... (much tobacco + much more tobacco)?[5]			
No school/elementary school	7	0(0-23)	0(0-0)
Lower secondary school	46	15(4-29)	13(8-18)
Intermediary secondary school	60	15(3-27)	16(11-20)
Higher vocational/university	67	27(15-39)	27(22-33)
**C. Changes in mental health factors**			
Compared to the period before the corona measures, I am having sleep... (more trouble sleeping + much more trouble sleeping)?[5]			
No school/elementary school	46	43(30-59)	40(33-46)
Lower secondary school	182	27(20-34)	28(25-32)
Intermediary secondary school	289	27(22-33)	26(24-29)
Higher vocational/university	455	19(16-23)	19(17-21)
Compared to the period before the corona measures, I am having stress... (more stressed + much more stressed)?[5]			
No school/elementary school	49	51(39-67)	47(40-53)

Lower secondary school	188	44(37-52)	46(43-50)
Intermediary secondary school	313	49(43-54)	47(44-50)
Higher vocational/university	480	38(33-42)	38(35-40)
Compared to the period before the corona measures, I am having lonely... (more lonely + much more lonely)?[5]			
No school/elementary school	46	33(20-48)	30(23-37)
Lower secondary school	178	31(24-38)	32(28-36)
Intermediary secondary school	284	29(24-35)	28(26-31)
Higher vocational/university	419	32(27-37)	32(30-34)
**D. Changes in access to non-COVID care**			
I am reluctant to go to the doctor for fear of catching the corona virus.... (agree + agree completely)?[5]			
No school/elementary school	55	27(15-42)	26(20-32)
Lower secondary school	214	28(21-35)	28(25-31)
Intermediary secondary school	277	20(14-26)	18(16-20)
Higher vocational/university	486	15(10-20)	14(13-16)
I am reluctant to allow caregivers into my home for fear of catching the corona virus.... (agree + agree completely)?[5]			
No school/elementary school	55	36(24-51)	30(25-35)
Lower secondary school	214	29(22-36)	29(26-32)
Intermediary secondary school	277	26(20-32)	24(22-27)
Higher vocational/university	486	14(10-19)	14(13-16)
I am currently being denied medical treatment due to the corona virus… (agree + agree completely)?[5]			
No school/elementary school	39	38(23-55)	32(26-39)
Lower secondary school	127	20(11-29)	19(15-22)
Intermediary secondary school	157	26(18-35)	23(20-27)
Higher vocational/university	229	16(10-23)	16(13-18)
I currently receive less professional care than before the corona crisis… (agree + agree completely)?[5]			
No school/elementary school	27	59(44-79)	61(55-68)
Lower secondary school	81	22(11-33)	23(18-28)
Intermediary secondary school	86	19(8-30)	20(15-24)
Higher vocational/university	123	18(9-27)	18(15-22)

[1] crude proportion (95% confidence intervals).
[2] proportion (95% confidence intervals) adjusted for age and sex. Proportions obtained using adj. prop package in R.
Adjustments were done according to the structure of the whole HELIUS COVID-19 sub-sample.
[3] total number of participants that reported as jobless (unemployed or job seeker) was **84**
adjusted proportions in age category were only adjusted for sex.
[4] total number of participants that reported as trouble with family income was **219**
[5] calculated only in those that apply
Within group totals do not always add to expected totals due to missings.

Appendix 8. Changes in multiple life domains due to the COVID-19 by occupation.

	Within group N	Crude	Age and sex adjusted
		% (95% CI)[1]	% (95% CI)[2]
**A. Changes in job status and income**			
Of those that are jobless, the cause is the coronavirus pandemic ... (yes)?[3]			
Elementary occupations	4	0(0-42)	0(0-0)
Lower occupations	23	22(9-39)	22(13-30)
Intermediary occupations	22	27(14-48)	27(18-37)
Higher occupations	15	33(13-56)	40(29-50)
Scientific occupations	3	0(0-56)	0(0-0)
Of those with trouble getting by family income, the cause is the coronavirus pandemic... (yes)?[4]			
Elementary occupations	22	72(56-93)	51(42-59)
Lower occupations	57	71(59-83)	64(59-70)
Intermediary occupations	64	66(55-79)	58(52-64)
Higher occupations	45	75(65-90)	67(61-73)
Scientific occupations	7	86(71-100)	89(87-90)
**B. Changes to less healthy behavior**			
Compared to the period before the corona measures, I exercise and participate in sports... (less + much less)?			
Elementary occupations	45	44(29-60)	42(36-48)
Lower occupations	180	47(39-55)	48(44-52)
Intermediary occupations	310	45(39-51)	45(42-48)
Higher occupations	322	40(34-46)	40(37-43)
Scientific occupations	145	39(30-47)	38(34-42)
Compared to the period before the corona measures, I eat... (less healthy + much less healthy)?			
Elementary occupations	45	10(0-23)	9(5-13)
Lower occupations	180	7(1-14)	7(5-9)
Intermediary occupations	310	11(6-16)	11(9-13)
Higher occupations	322	8(4-12)	8(6-9)
Scientific occupations	145	8(2-15)	9(6-11)
Compared to the period before the corona measures, I drink... (much alcohol + much more alcohol)?[5]			
Elementary occupations	9	0(0-39)	0(0-0)
Lower occupations	72	6(0-17)	7(4-10)
Intermediary occupations	142	11(4-20)	10(8-13)
Higher occupations	204	15(8-21)	15(12-17)
Scientific occupations	117	12(4-21)	12(9-15)
Compared to the period before the corona measures, I smoke... (much tobacco + much more tobacco)?[5]			
Elementary occupations	6	0(0-50)	0(0-0)
Lower occupations	41	7(0-21)	6(3-10)
Intermediary occupations	63	22(11-35)	21(16-26)
Higher occupations	38	24(11-42)	28(22-33)
Scientific occupations	22	27(9-50)	21(14-28)
**C. Changes in mental health factors**			
Compared to the period before the corona measures, I am having sleep... (more trouble sleeping + much more trouble sleeping)?[5]			

Elementary occupations	44	32(18-47)	32(26-37)
Lower occupations	173	29(22-36)	29(25-32)
Intermediary occupations	262	23(17-28)	22(20-25)
Higher occupations	268	21(16-27)	22(19-24)
Scientific occupations	145	18(12-24)	18(14-21)
Compared to the period before the corona measures, I am having stress... (more stressed + much more stressed)?[5]			
Elementary occupations	50	46(34-62)	47(41-54)
Lower occupations	180	42(34-50)	42(38-46)
Intermediary occupations	281	46(40-52)	45(42-48)
Higher occupations	283	43(37-50)	43(40-46)
Scientific occupations	149	30(23-39)	31(27-34)
Compared to the period before the corona measures, I am having lonely... (more lonely + much more lonely)?[5]			
Elementary occupations	44	23(9-37)	23(17-30)
Lower occupations	169	32(25-39)	31(28-35)
Intermediary occupations	255	29(24-35)	29(26-32)
Higher occupations	243	33(27-39)	32(29-35)
Scientific occupations	137	30(23-38)	31(27-35)
**D. Changes in access to non-COVID care**			
I am reluctant to go to the doctor for fear of catching the corona virus.... (agree + agree completely)?[5]			
Elementary occupations	42	43(29-59)	43(34-52)
Lower occupations	205	26(20-34)	25(22-28)
Intermediary occupations	272	19(14-26)	19(16-21)
Higher occupations	299	13(7-19)	13(11-15)
Scientific occupations	137	13(5-23)	13(10-16)
I am reluctant to allow caregivers into my home for fear of catching the corona virus…. (agree + agree completely)?[5]			
Elementary occupations	42	43(29-59)	44(35-52)
Lower occupations	205	29(22-36)	28(25-31)
Intermediary occupations	272	25(18-31)	25(22-28)
Higher occupations	299	13(7-19)	13(11-15)
Scientific occupations	137	13(5-22)	13(10-16)
I am currently being denied medical treatment due to the corona virus… (agree + agree completely)?[5]			
Elementary occupations	24	42(25-65)	46(39-53)
Lower occupations	114	29(20-39)	30(25-34)
Intermediary occupations	162	15(7-23)	14(11-17)
Higher occupations	141	18(9-26)	17(13-20)
Scientific occupations	62	16(5-30)	18(13-23)
I currently receive less professional care than before the corona crisis… (agree + agree completely)?[5]			
Elementary occupations	21	43(24-66)	36(27-44)
Lower occupations	61	25(13-39)	25(20-31)
Intermediary occupations	92	15(5-27)	15(11-19)
Higher occupations	73	21(10-33)	21(16-26)
Scientific occupations	33	21(6-40)	24(16-32)

[1] crude proportion (95% confidence intervals).

[2] proportion (95% confidence intervals) adjusted for age and sex. Proportions obtained using adj. prop package in R. Adjustments were done according to the structure of the whole HELIUS COVID-19 sub-sample.

[3] total number of participants that reported as jobless (unemployed or job seeker) was **84**
adjusted proportions in age category were only adjusted for sex.
[4] total number of participants that reported as trouble with family income was **219**
[5] calculated only in those that apply
Within group totals do not always add to expected totals due to missings.

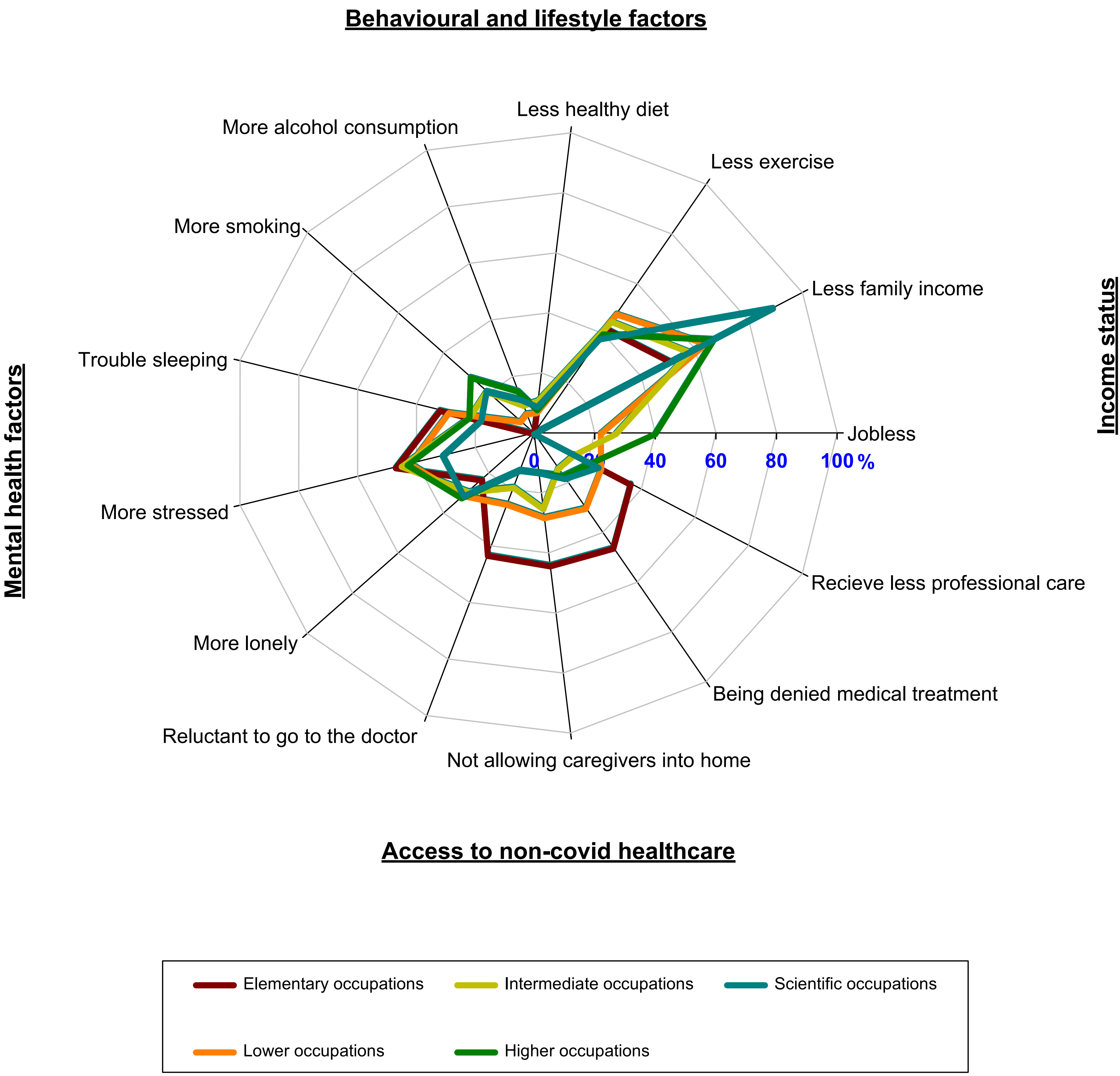
Behavioural and lifestyle factors
Income status
Mental health factors
Access to non-covid healthcare
Less healthy diet
Less exercise
Less family income
Jobless
Recieve less professional care
Being denied medical treatment
Not allowing caregivers into home
Reluctant to go to the doctor
More lonely
More stressed
Trouble sleeping
More smoking
More alcohol consumption
0
40
60
80
100 %
Elementary occupations
Intermediate occupations
Scientific occupations
Lower occupations
Higher occupations
